# Genetic background of obesity: a case-control study

**DOI:** 10.1007/s42000-026-00790-3

**Published:** 2026-06-02

**Authors:** Konstantinos Kalesis, Paris Roidοs, Vaios Nikolopoulos, Niki Katsiki, Kalliopi Kotsa, Theoharis Koufakis

**Affiliations:** 1 Department of Medicine, School of Health Sciences, Aristotle University of Thessaloniki, Thessaloniki, Greece; 2 Molecular Biology Laboratory DNA Therapeutics, Thessaloniki, Greece; 3 Diagnostic Laboratory Istodierevnitiki, Thessaloniki, Greece; 4 Department of Veterinary Medicine, School of Health Sciences, University of Thessaly, Karditsa, Greece; 5 Department of Nutritional Sciences and Dietetics, International Hellenic University, Thessaloniki, Greece; 6 School of Medicine, European University of Cyprus, Nicosia, Cyprus; 7 Department of Endocrinology and Metabolism, First Department of Internal Medicine, Aristotle University of Thessaloniki, AHEPA University Hospital, Thessaloniki, Greece; 8 Second Department of Internal Medicine, Aristotle University of Thessaloniki, Hippokration General Hospital, Thessaloniki, Greece

**Keywords:** Obesity, Type 2 diabetes, Gene polymorphisms, TCF7L2, FTO, PPARG, HbA1c, Hardy-Weinberg equilibrium, Personalized medicine

## Abstract

**Purpose:**

The rising prevalence of obesity and type 2 diabetes mellitus (T2DM) constitutes a major global public health concern. Increasing evidence indicates that genetic polymorphisms may modulate the risk of T2DM in individuals with obesity, providing valuable insight into the biological pathways that connect these interrelated disorders. A better understanding of the genetic determinants underlying obesity-associated T2DM could enhance disease characterization and inform risk stratification strategies. The genetic architecture linking obesity and T2DM has, however, not yet been fully elucidated.

**Materials and methods:**

In this case-control study, we investigated the distribution of the TCF7L2 rs7903146, FTO rs9939609, and PPARG rs1801282 polymorphisms in 257 Greek adults with obesity (BMI > 30 kg/m²), including 136 individuals without T2DM and 121 with T2DM. The primary prespecified analysis applied unadjusted additive logistic regression to estimate per-allele odds ratios (ORs) for T2DM risk, while dominant and recessive inheritance models were evaluated as secondary analyses. Hardy-Weinberg equilibrium was assessed in the control group using an exact test. In participants without diabetes, genotype distributions were further compared across clinically defined HbA1c categories based on American Diabetes Association thresholds. The study was conducted and reported in accordance with STREGA guidelines.

**Results:**

Genotype distributions differed significantly between cases and controls for all three loci. In the additive model, each additional T allele at TCF7L2 rs7903146 was associated with higher odds of T2DM (OR 2.66, 95% CI 1.92–3.68; *p* < 0.001). Similar associations were observed for FTO rs9939609 (OR 1.52, 95% CI 1.14–2.03; *p* = 0.005) and PPARG rs1801282 (OR 1.98, 95% CI 1.41–2.77; *p* < 0.001). No statistically significant association was detected between the examined variants and HbA1c category in the non-diabetic group. In controls, all three loci deviated from HWE, with marked heterozygote deficits for TCF7L2 and FTO.

**Conclusions:**

TCF7L2 rs7903146 and FTO rs9939609 were associated with T2DM among individuals with obesity in this cohort, whereas PPARG rs1801282 showed an association in the opposite direction from that reported in much of the prior literature. These findings should be interpreted cautiously because the regression models were unadjusted, while the control-group HWE deviations raise concerns regarding assay or sampling validity. Larger, well-characterized studies with participant-level covariate data and expanded quality-control procedures are required.

## Introduction

Diabetes mellitus does not follow a single pattern of inheritance and comprises monogenic as well as polygenic forms. Type 2 diabetes mellitus (T2DM), the most common form, arises from a complex interaction between genetic susceptibility and environmental exposure 1–3. Obesity is one of the principal modifiers of that risk, and the coexistence of obesity and T2DM substantially amplifies the long-term burden of cardiovascular, renal, neurologic, and ophthalmologic complications^2^,^3^. Collectively, these complications gradually lead to a substantial reduction in quality of life 2˒^3^.

Genetic variants associated with T2DM may be present in both healthy individuals and affected patients, with frequencies ranging from common to rare, without necessarily resulting in clinical disease manifestation. Thus, these polymorphisms are not direct causal determinants of T2DM but rather genetic risk factors that may increase susceptibility to its development 1.

Genome-wide association studies (GWAS) have substantially expanded current knowledge of the genetic architecture of T2DM. These large-scale analyses are designed to identify common genetic variants associated with the onset, progression, or specific phenotypic traits of complex diseases 4. Among these variants, single nucleotide polymorphisms (SNPs), defined as substitutions of a single nucleotide within the DNA sequence, have been associated not only with T2DM but also with related metabolic conditions, including insulin resistance 5–7.

Since 2017, thirteen novel susceptibility loci have been identified and categorized into three major groups of genes associated with T2DM 8. The first group, including GIPR, C2CDC4A, GCK, CDKAL1, TCF7L2, GLIS3, THADA, IGF2BP2, and DGKB, is primarily involved in insulin secretion and processing. The second group, comprising PPARG, KLF14, and IRS1, is related to insulin action. The third group, including NRXN3, CMIP, APOE, and MC4R, has been associated with body mass index (BMI) and lipid metabolism. Collectively, these findings underscore the contribution of a complex genetic background and multiple biological pathways to the pathophysiology of T2DM 8. A large number of genes have been proposed as potential genetic predictors of T2DM risk, including KLF14, KCNQ1, DUSP9, FTO, HNF4A, IGFBP2, CDKN2A/B, TCF7L2, KCNJ11, DNAJC3, PGC-1α, ADIPOQ, CDKAL1, POMC, PPARγ2, and SLC30A8 2. Among the most consistently implicated loci is TCF7L2, particularly the rs7903146 variant, which has shown reproducible associations with T2DM across populations^9^,^10^. Variants in the FTO (fat mass and obesity-associated gene), including rs9939609, are strongly associated with adiposity and may also influence diabetes risk through obesity-related and obesity-independent pathways^11^,^12^. The common Pro12Ala variant in PPARG (rs1801282) has often been linked to reduced T2DM risk in large meta-analyses, although the magnitude and direction of association appear to vary across populations and study designs^13^,^14^.

The present case-control study was designed to compare the frequency of TCF7L2 rs7903146, FTO rs9939609, and PPARG rs1801282 polymorphisms between two groups of Greek adults with obesity, namely, individuals without T2DM and individuals with T2DM. A secondary objective was to examine whether these variants were associated with HbA1c levels within the non-diabetic obese group. The manuscript has been revised in accordance with the STrengthening the REporting of Genetic Association studies (STREGA) recommendations^15^.

## Materials and methods

### Study population

This case-control study included 257 adults with obesity (BMI > 30 kg/m²) who were recruited between 20 May, 2023 and 20 May, 2024. After oral and written informed consent had been obtained, blood samples were collected in anonymized and coded form for molecular analysis. The study protocol, data-handling procedures, and informed-consent documents were approved by the Ethics Committee of Aristotle University of Thessaloniki (approval no. 84/2023, December 20, 2023; session no. 2/12.12.2023). Participants were divided into two groups. The control group comprised 136 individuals with obesity but without T2DM. The case group comprised 121 individuals with obesity and established T2DM. All participants were of Greek Caucasian ancestry according to the study record. Individuals with known monogenic diabetes or severe comorbid conditions were excluded in order to focus on typical obesity-associated T2DM.

## Clinical evaluation

Routine clinical evaluation at the recruitment sites included age, sex, medical history, and anthropometric assessment. However, the de-identified analytical dataset made available for the present statistical analyses did not contain participant-level values for age, sex, or BMI. These covariates were available only as group-level summaries (means, ranges, and proportions), which allowed descriptive reporting by group but did not permit participant-level regression adjustment. This distinction is important for interpretation of the revised analyses. Accordingly, age, sex, and BMI are reported descriptively in Table 1 as aggregate characteristics only, and the genotype-T2DM association models are presented as unadjusted models.

Genomic DNA was isolated from whole blood using a CE-IVD-certified commercial extraction kit according to the manufacturer’s instructions. Genotyping was performed at the accredited molecular biology laboratory “DNA Therapeutics” (Pylaia, Thessaloniki, Greece) by real-time polymerase chain reaction using the rhAmp SNP Genotyping System (Integrated DNA Technologies). The examined polymorphisms were TCF7L2 rs7903146 (CC, CT, TT), FTO rs9939609 (AA, AT, TT), and PPARG rs1801282 (CC, CG, GG).

Quality control procedures included assessment of genotype call rates, duplicate genotyping of approximately 10% of samples, and HWE testing in controls. All three assays achieved a 100% call rate, and duplicate genotyping showed 100% concordance. HWE was evaluated using an exact test, consistent with standard recommendations for genotype quality assessment^16^,^17^.

In parallel, as a secondary analysis with genetic testing, glycated hemoglobin (HbA1c) levels were measured for participants in the obesity without T2DM group (controls). HbA1c was measured in EDTA-anticoagulated whole blood using high-performance liquid chromatography at certified clinical laboratories selected by the participants as part of routine clinical care. HbA1c was categorized as normal glycemia (4.0-5.6) or prediabetes (5.7–6.4) according to ADA diagnostic thresholds^19^. By definition, participants in the control group did not have HbA1c values in the diabetic range.

### Statistical analysis

Categorical variables are presented as counts and percentages. Quantitative variables are presented only in the format supported by the available data: age as mean (range), BMI as group mean, and HbA1c as mean ± SD where those summary statistics were available. Because participant-level age and BMI data were not available, formal normality assessment and estimation of standard deviations for those variables were not possible. Where individual-level HbA1c data were available in controls, values were used for category-based analysis rather than continuous regression modeling.

Genotype frequencies were compared between groups using the chi-square test. In the prespecified primary association analysis, unconditional logistic regression was used, with T2DM status as the dependent variable and genotype coded additively (per risk allele) as the predictor. Dominant and recessive models were examined as secondary analyses. These models were intentionally left unadjusted because participant-level covariate data for age, sex, and BMI were not available. Associations between genotype and HbA1c levels in the non-diabetic group were evaluated using the chi-square test. All tests were two-sided with a significance level of 0.05. Analyses were performed using IBM SPSS Statistics version 26. The revised report was prepared in accordance with STREGA guidance^19^.

## Results

Aggregate participant characteristics are summarized in Table 1. The two groups were similar with respect to sex distribution and mean BMI, while the T2DM group was modestly older on average. As expected, mean HbA1c was higher in the T2DM group.

**Table 1:** Participant characteristics

Characteristics	Obesity without T2DM (*n* = 136)	Obesity with T2DM (*n* = 121)
Age, years	50 (18–67)	54 (18–72)
Female sex, n (%)	74 (54.4%)	65 (53.7%)
Male sex, n (%)	62 (45.6%)	56 (46.3%)
BMI, kg/m²	33.2	32.8
HbA1c	5.4 ± 0.4	7.8 ± 1.5

Age and *BMI* were available only as group-level summaries; therefore, age is reported as mean (range), *BMI* as group mean, and no participant-level standard deviations or formal between-group tests could be derived for these variables. HbA1c is presented as mean ± SD as available from the study summaries

All 257 participants were successfully genotyped for all three loci. Duplicate genotyping of approximately 10% of samples showed complete concordance. In the control group, the exact HWE test indicated marked deviation from equilibrium for TCF7L2 rs7903146 and FTO rs9939609, with a milder deviation for PPARG rs1801282. In each instance, the deviation reflected a deficit of heterozygotes relative to HWE expectations (Table 2).

**Table 2:** Hardy-Weinberg equilibrium (HWE) assessment in controls (*n* = 136)

SNP	Observed heterozygotes, *n* (%)	Expected heterozygotes, *n* (%)	Exact HWE *p*-value	Interpretation
TCF7L2 rs7903146	19 (14.0%)	40.2 (29.5%)	< 0.001	Marked heterozygote deficit
FTO rs9939609	11 (8.1%)	49.4 (36.3%)	< 0.001	Marked heterozygote deficit
PPARG rs1801282	36 (26.5%)	46.0 (33.8%)	0.019	Mild heterozygote deficit

Expected heterozygote counts were calculated from control-group allele frequencies. Exact *p* values were derived from the observed control genotype counts 16

Genotype distributions differed significantly between individuals with obesity alone and those with obesity plus T2DM for all three loci (Table 3). The most pronounced difference was observed for TCF7L2 rs7903146, for which the TT genotype was much more frequent in T2DM cases than in controls.

**Table 3:** Distribution of genotypes according to study group (*n* = 257)

Polymorphism	Genotype	Obesity without T2DM (*n* = 136)	Obesity with T2DM (*n* = 121)	χ² test *p*-value
TCF7L2 rs7903146	CC (wild-type)	102 (75.0%)	48 (39.7%)	< 0.001
	CT (heterozygous)	19 (14.0%)	22 (18.2%)	
	TT (homozygous)	15 (11.0%)	51 (42.1%)	
FTO rs9939609	AA (wild-type)	98 (72.1%)	64 (52.9%)	0.006
	AT (heterozygous)	11 (8.1%)	19 (15.7%)	
	TT (homozygous)	27 (19.9%)	38 (31.4%)	
PPARγ rs1801282	CC (wild-type)	89 (65.4%)	57 (47.1%)	< 0.001
	CG (heterozygous)	36 (26.5%)	29 (24.0%)	
	GG (homozygous)	11 (8.1%)	35 (28.9%)	

Overall *p* values are based on chi-square tests comparing the full genotype distributions between cases and controls. Abbreviation: *T2DM* type 2 diabetes mellitus

**Fig. 1:**
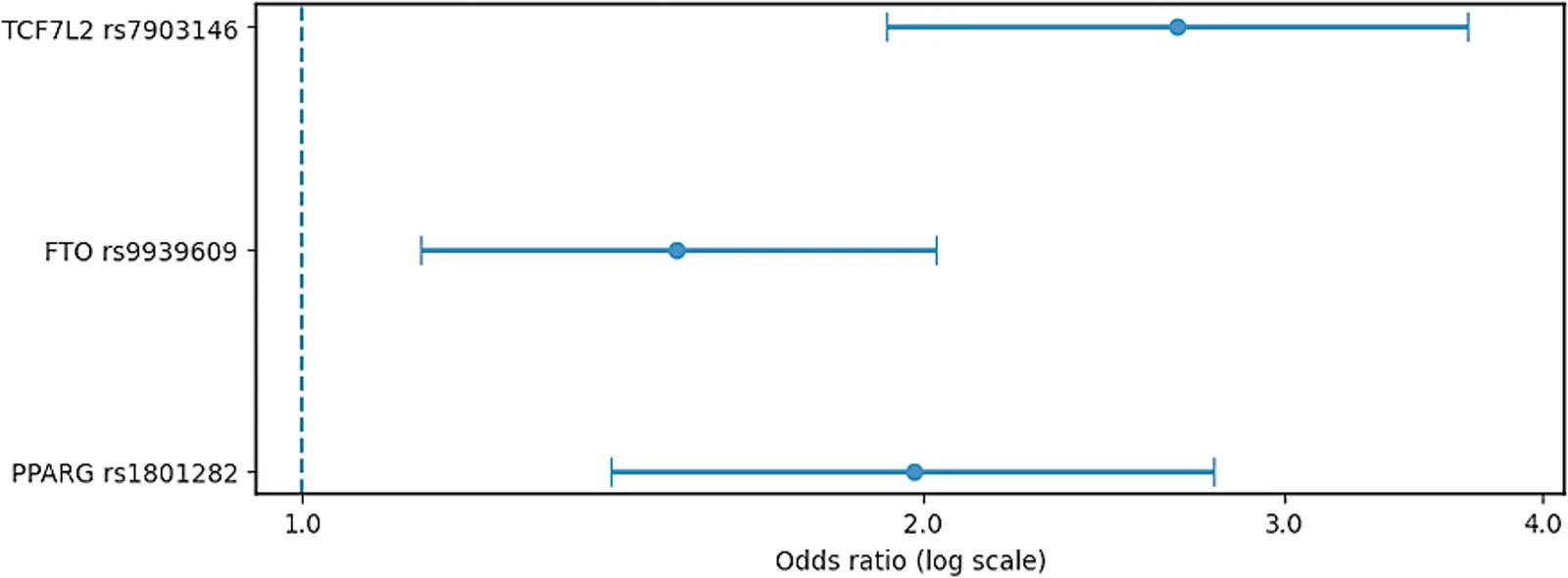
Forest plot of unadjusted additive-model odds ratios for T2DM. Error bars represent 95% confidence intervals

In the primary additive model, each additional T allele at TCF7L2 rs7903146 was associated with higher odds of T2DM (OR 2.66, 95% CI 1.92–3.68; *p* < 0.001). The FTO rs9939609 T allele and the PPARG rs1801282 G allele were also associated with higher T2DM odds in additive models. Similar patterns were observed in the dominant and recessive models (Table 4; Fig. 1).

**Table 4:** Logistic-regression analyses for the association between genotype and T2DM

Polymorphism (SNP)	Genetic model	Unadjusted OR (95% CI)	Unadjusted *p*-value	Notes
TCF7L2 rs7903146	Additive (per T allele)	2.66 (1.92–3.68)	p < 0.001	Secondary model
	Dominant (CT + TT vs. CC)	4.56 (2.68–7.77)	p < 0.001	Secondary model
	Recessive (TT vs. CC + CT)	5.88 (3.08–11.22)	p < 0.001	Primary model; unadjusted
				Secondary model
FTO rs9939609	Additive (per T allele)	1.52 (1.14–2.03)	p = 0.005	Secondary model
	Dominant (AT + TT vs. AA)	2.30 (1.37–3.85)	p = 0.002	Primary model; unadjusted
	Recessive (TT vs. AA + AT)	1.85 (1.05–3.27)	p = 0.035	Secondary model
				Secondary model
PPARγ rs1801282	Additive (per G allele)	1.98 (1.41–2.77)	p < 0.001	Secondary model
	Dominant (CG + GG vs. CC)	2.13 (1.29–3.51)	p = 0.003	Secondary model
	Recessive (GG vs. CC + CG)	4.62 (2.23–9.61)	p < 0.001	Primary model; unadjusted

All models are unadjusted because participant-level age, sex, and *BMI* data were not available for regression modeling. The additive (per-allele) model is the prespecified primary analysis; dominant and recessive models are secondary

Within the non-diabetic obese group, no statistically significant difference in genotype distribution was observed between participants with normal HbA1c and those with prediabetes-range HbA1c (Table 5). These findings do not support an association between the examined variants and HbA1c category in this subgroup.

**Table 5:** Results of the analysis for association with HbA1c

Polymorphism (SNP)	Genotype	HbA1c < 5.7 (*n* = 47)	HbA1c 5.7–6.4 (*n* = 89)	Chi-square test, *p*-value
TCF7L2 rs7903146	CC	36 (76.6%)	66 (74.2%)	p = 0.786
	CT	7 (14.9%)	12 (13.5%)	
	TT	4 (8.5%)	11 (12.4%)	
FTO rs9939609	AA	38 (73.1%)	60 (71.4%)	p = 0.978
	AT	4 (7.7%)	7 (8.3%)	
	TT	10 (19.2%)	17 (20.2%)	
PPARγ rs1801282	CC	31 (63.3%)	58 (66.7%)	p = 0.912
	CG	14 (28.6%)	22 (25.3%)	
	GG	4 (8.2%)	7 (8.0%)	

*P* values are based on chi-square tests comparing genotype distributions between HbA1c categories

## Discussion

This analysis identified meaningful associations between T2DM status and all three examined loci in a cohort of Greek adults with obesity. The strongest signal was observed for TCF7L2 rs7903146, followed by PPARG rs1801282 and FTO rs9939609 in additive models. Overall, the direction of association for TCF7L2 and FTO is in line with a substantial body of literature linking these loci to beta-cell dysfunction, obesity-related traits, or both 9-12-20-23 and it supports the biological rationale for focusing on these variants in obesity-related T2DM.

The finding for PPARG rs1801282 is particularly interesting. Much of the literature, including a large Human Genome Epidemiology review and meta-analysis, has suggested that the Ala12 (G) allele is associated with reduced T2DM risk overall^13^,^14^. In the present cohort, however, the G allele was more frequent among T2DM cases. Rather than diminishing the value of the result, this difference may point to the importance of cohort composition, obesity-specific metabolic context, population characteristics, or other methodological factors when interpreting PPARG associations. As such, this observation adds nuance to the current evidence base and provides a useful direction for future confirmatory work.

The HWE findings merit careful consideration and are best viewed as an important quality control and interpretive context for the present results. Exact testing showed substantial heterozygote deficits for TCF7L2 and FTO and a milder deficit for PPARG. Departure from HWE in control samples may arise from several sources, including assay problems, genotyping error, non-random sampling, inbreeding, or population stratification 16-18. Hosking et al. demonstrated that HWE testing can identify problematic assays in a meaningful fraction of high-throughput genotyping datasets^17^. Likewise, principal-components approaches have become standard for detecting and correcting population structure in large-scale association studies because ancestry differences can produce spurious case-control associations^18^. Although the present study is a focused candidate-gene analysis rather than a genome-wide study, these considerations provide an appropriate framework for interpreting the reported associations with due scientific care.

At the same time, the duplicate-genotyping concordance rate of 100% is reassuring and supports the technical reliability of the laboratory procedures that were possible within this study. Given that the duplicate subset was relatively small (approximately 10%), broader confirmatory testing would further strengthen confidence in future work; nevertheless, the available quality control data support retaining these loci in an exploratory analysis. In this context, the present findings are best regarded as informative and hypothesis-generating, offering a sound basis for larger studies with expanded laboratory validation and, where possible, ancestry-informative analyses.

The unadjusted logistic-regression models should likewise be interpreted in the context of data availability. Although age, sex, and BMI were clinically recorded, only group-level summaries were available to the investigators for the present analysis. This allowed a transparent description of the cohort and a clear exploratory comparison between groups, but it did not permit participant-level covariate adjustment. Accordingly, the reported ORs should be interpreted as unadjusted estimates, particularly in view of the approximately 4-year difference in mean age between cases and controls. Transparent reporting of these analytical constraints is explicitly recommended by STREGA^15^.

The analysis of HbA1c category in controls did not show statistically significant genotype differences. This result should be understood as indicating that no association was detected in this sample using clinically defined categories. Given the modest sample size and the categorical treatment of HbA1c, subtle genetic effects may not have been captured and this question remains worthy of further investigation in larger datasets.

Several features nevertheless strengthen the contribution of the study, including the clinically well-defined case-control design, the ethnically homogeneous Greek cohort, complete genotyping call rates, and perfect concordance in duplicate testing within the available quality-control subset. In addition, published evidence from Greek populations remains limited. Although smaller, ethnically focused studies cannot replace large multicenter consortia, they can provide valuable local data, generate biologically relevant hypotheses, and highlight population-specific patterns that deserve further study 24-29.

### Limitations

(i) As a case-control study, the design cannot estimate incidence and remains vulnerable to selection and survivor biases. (ii) The sample size was modest, increasing the risk of both false-positive and false-negative findings. (iii) HbA1c measurements were obtained through routine clinical laboratories, introducing the possibility of inter-laboratory measurement heterogeneity. (iv) Participant-level age, sex, and BMI data were not available in the analytical dataset; therefore, regression models could not be adjusted for these potential confounders. (v) All three loci deviated from HWE in controls, particularly TCF7L2 rs7903146 and FTO rs9939609, which raises concerns regarding genotyping validity, sampling effects, or population structure and may affect the reliability of the reported associations.

## Conclusions

In this cohort of individuals with obesity, TCF7L2 rs7903146 and FTO rs9939609 were associated with higher odds of T2DM, while PPARG rs1801282 showed an association in a direction that differs from that indicated by much of the published literature. Taken together, these findings reinforce the biological relevance of genetic susceptibility in obesity-related T2DM and add useful data from a Greek population that remains underrepresented in the literature. The results should be interpreted within the methodological context described above, while confirmation in larger, comprehensively characterized cohorts will be important. Even so, the present study provides a constructive foundation for future work in genetic risk assessment and personalized approaches to metabolic disease.
